# Management of Medullary Thyroid Cancer: Patterns of Recurrence and Outcomes of Reoperative Surgery

**DOI:** 10.1093/oncolo/oyad232

**Published:** 2023-08-26

**Authors:** Alexander J Papachristos, Laura E Nicholls, Robert Mechera, Ahmad M Aniss, Bruce Robinson, Roderick Clifton-Bligh, Anthony J Gill, Diana Learoyd, Stan B Sidhu, Anthony Glover, Leigh Delbridge, Mark Sywak

**Affiliations:** Department of Endocrine Surgery, Endocrine Surgical Unit, Royal North Shore Hospital, Northern Sydney Local Health District, Sydney, NSW, Australia; Department of Surgery, Northern Clinical School, Sydney Medical School, Faculty of Medicine and Health, University of Sydney, Sydney, NSW, Australia; Department of Endocrine Surgery, Endocrine Surgical Unit, Royal North Shore Hospital, Northern Sydney Local Health District, Sydney, NSW, Australia; Department of Endocrine Surgery, Endocrine Surgical Unit, Royal North Shore Hospital, Northern Sydney Local Health District, Sydney, NSW, Australia; Department of Endocrine Surgery, Endocrine Surgical Unit, Royal North Shore Hospital, Northern Sydney Local Health District, Sydney, NSW, Australia; Department of Surgery, Northern Clinical School, Sydney Medical School, Faculty of Medicine and Health, University of Sydney, Sydney, NSW, Australia; Department of Surgery, Northern Clinical School, Sydney Medical School, Faculty of Medicine and Health, University of Sydney, Sydney, NSW, Australia; Department of Endocrinology, Royal North Shore Hospital, Sydney, NSW, Australia; Department of Cancer Diagnosis and Pathology, Cancer Diagnosis and Pathology Group, Kolling Institute of Medical Research, Royal North Shore Hospital, Sydney, NSW, Australia; Department of Surgery, Northern Clinical School, Sydney Medical School, Faculty of Medicine and Health, University of Sydney, Sydney, NSW, Australia; Department of Endocrinology, Royal North Shore Hospital, Sydney, NSW, Australia; Department of Cancer Diagnosis and Pathology, Cancer Diagnosis and Pathology Group, Kolling Institute of Medical Research, Royal North Shore Hospital, Sydney, NSW, Australia; Department of Surgery, Northern Clinical School, Sydney Medical School, Faculty of Medicine and Health, University of Sydney, Sydney, NSW, Australia; Department of Anatomical Pathology, NSW Health Pathology, Royal North Shore Hospital, Sydney, NSW, Australia; GenesisCare North Shore Health Hub Tower A, NSW, Australia; Department of Endocrine Surgery, Endocrine Surgical Unit, Royal North Shore Hospital, Northern Sydney Local Health District, Sydney, NSW, Australia; Department of Surgery, Northern Clinical School, Sydney Medical School, Faculty of Medicine and Health, University of Sydney, Sydney, NSW, Australia; Department of Endocrine Surgery, Endocrine Surgical Unit, Royal North Shore Hospital, Northern Sydney Local Health District, Sydney, NSW, Australia; Department of Surgery, Northern Clinical School, Sydney Medical School, Faculty of Medicine and Health, University of Sydney, Sydney, NSW, Australia; Department of Cancer Research, The Kinghorn Cancer Centre, Garvan Institute of Medical Research, St. Vincent’s Clinical School, Faculty of Medicine, University of New South Wales, Sydney, NSW, Australia; Department of Endocrine Surgery, Endocrine Surgical Unit, Royal North Shore Hospital, Northern Sydney Local Health District, Sydney, NSW, Australia; Department of Endocrine Surgery, Endocrine Surgical Unit, Royal North Shore Hospital, Northern Sydney Local Health District, Sydney, NSW, Australia; Department of Surgery, Northern Clinical School, Sydney Medical School, Faculty of Medicine and Health, University of Sydney, Sydney, NSW, Australia

**Keywords:** medullary thyroid cancer, thyroidectomy, lymph node dissection, thyroid, recurrence, endocrine surgery

## Abstract

**Background:**

There remains uncertainty regarding the optimal extent of initial surgery and management of recurrent disease in medullary thyroid cancer (MTC). We aim to describe the patterns of disease recurrence and outcomes of the reoperative surgery in a cohort of consecutively treated patients at a specialized tertiary referral center.

**Patients and Methods:**

A retrospective cohort study of 235 surgically treated patients with MTC at a tertiary referral center was performed using prospectively collected data.

**Results:**

In the study period 1986-2022, 235 patients underwent surgery for MTC. Of these, 45 (19%) patients had reoperative surgery for cervical nodal recurrence at a median (range) 2.1 (0.3-16) years following the index procedure. After a median follow-up of 4 years, 38 (84%) patients remain free of structural cervical recurrence, although 15 (33%) underwent 2 or more reoperative procedures. No long-term complications occurred after reoperative surgery. Local cervical recurrence was independently predicted by pathologically involved nodal status (OR 5.10, *P* = .01) and failure to achieve biochemical cure (OR 5.0, *P* = .009). Local recurrence did not adversely affect overall survival and was not associated with distant recurrence (HR 0.93, *P* = .83). Overall survival was independently predicted by high pathological grade (HR 10.0, *P* = .002) and the presence of metastatic disease at presentation (HR 8.27, *P* = 0018).

**Conclusion:**

Loco-regional recurrence in MTC does not impact overall survival, or the development of metastatic disease, demonstrating the safety of the staged approach to the clinically node-negative lateral neck. When recurrent disease is technically resectable, reoperative surgery can be undertaken with minimal morbidity in a specialized center and facilitates structural disease control.

## Teaser

Uncertainty remains regarding the optimal extent of initial surgery and management of recurrent disease in medullary thyroid cancer. This article reports patterns of disease recurrence and outcomes of reoperative surgery in a cohort of consecutively treated patients.

**Implications for Practice**

This study describes the patterns of disease recurrence in medullary thyroid cancer (MTC), and the outcomes associated with reoperative surgery. Local recurrence requiring reoperative surgery occurs in 19% of the patients at extended follow-up but does not affect survival or the development of metastatic disease. This adds to the growing body of evidence that prophylactic lateral neck dissection is not indicated in clinically N0 patients, and that reoperative surgery can be safely performed in specialized centers to provide structural disease control.

## Introduction

Medullary thyroid cancer (MTC) is a rare neuroendocrine tumor of parafollicular (C cells) and accounts for 2% of thyroid malignancies; however, it is responsible for 8% of thyroid cancer-related deaths.^1^ Most medullary thyroid carcinomas are sporadic and typically present between the fourth and sixth decades of life, often with nodal involvement or distant metastatic disease.^2,3^ Approximately, 15%-20% of MTC cases are familial and associated with a germline mutation in the rearranged during transfection (*RET*) protooncogene (multiple endocrine neoplasia type 2, MEN2).^4^ Because of its propensity for vascular invasion, lymph node involvement^5,6^ and distant metastasis, MTC is notoriously difficult to cure. However, it is often associated with an indolent clinical course and may take years or decades to progress, with an overall survival of approximately 70%-80% at 10 years.^7,8^ Therefore, clinicians must strike an effective balance between achieving adequate oncological management and minimizing surgical morbidity.

In the treatment of MTC with curative intent, guidelines recommend total thyroidectomy and bilateral central compartment dissection, with additional lateral compartmental dissection if there is evidence of lateral nodal involvement.^9^ For patients with MTC with no clinical evidence of lateral lymph node metastases, there is a lack of consensus to guide surgical management^10-12^ and significant variation in clinical practice exists.^13-15^ Historically, due to the lack of effective adjuvant therapies for MTC and the inaccuracy associated with staging ultrasound (US)^16^ and intraoperative nodal assessment,^5^ aggressive surgical management of the lymph node basins was often favored.^7,8^ However, with the increasing diagnostic sensitivity of high-resolution US and CT,^17,18^ as well as the emergence of effective-targeted therapies for patients with metastatic disease,^19-21^ there is a trend to de-escalating surgical treatment of the clinically negative lateral neck compartments.^22^ Aggressive lymphadenectomy has not been demonstrated to result in a long-term survival benefit^14,23,24^ despite resulting in lower postoperative calcitonin levels.^25^

Patients with a normal (<10 pg/mL) postoperative calcitonin level achieve “biochemical cure” and have a 97% 10-year survival,^26^ with a 3% recurrence risk.^27^ However, elevated serum calcitonin after seemingly adequate surgical excision is a common clinical problem, and the approach to further investigation and surgical management is variable. In particular, the decision to consider further nodal dissection must weigh the often indolent course of the disease, the risk of distant failure, and potential treatment-related morbidity.^28,29^ Predicting local recurrence, distant metastases, and survival is based on a combination of clinical and pathological factors, and the predictive utility of individual parameters varies between reports.^30-34^ Recently, a pathological grading system has been described and validated as an independent predictor of survival in MTC.^35^

We aim to describe the patterns of recurrent disease in patients with MTC and explore predictive clinical and pathological factors. In addition, we aim to describe the safety and outcomes of reoperative surgery for the management of locoregional recurrence.

## Patients and Methods

### Study Design and Study Population

A retrospective cohort study was undertaken. The study population included patients treated surgically for MTC in the period 1986 and 2022 at a single tertiary referral endocrine surgery unit. A comprehensive thyroid cancer database, which contains prospectively collected preoperative, operative, and follow-up data, was utilized to identify cases. Approval to access these data and perform the study was obtained from the Northern Sydney Local Health District Ethics Committee (reference 2020/ETH02787).

### Initial Surgical Management

Patients with clinically apparent tumors and a clear preoperative diagnosis of MTC were treated with a total thyroidectomy and bilateral central compartment lymph node dissection (CLND). Patients with atypical preoperative cytology underwent a staged completion thyroidectomy and consideration of bilateral CLND once histology was confirmed. The decision to perform lateral compartmental dissection was individualized and made by the treating surgeon based on results of serum calcitonin and imaging. The extent of lateral nodal dissection was defined according to accepted definitions^36^ and described according to the levels dissected. Our routine approach to the lateral neck in MTC includes dissection of levels II, III, IV, and Vb.

### Histopathological Analysis

A specialist endocrine pathologist confirmed the diagnosis of MTC and provided a structured report on all tumor specimens using SDs.^37,38^ MTC grade was classified according to The International Medullary Thyroid Cancer Grading System, with high-grade defined as a tumor demonstrating one of: tumor necrosis, mitotic rate ≥5 per 2 mm^3^, or Ki67 proliferation index ≥5%. Other pathological features included tumor size, vascular invasion, extrathyroidal extension (ETE), nodal status, and the presence of extranodal extension. Tumors were staged according to the American Joint Committee on Cancer (AJCC) eighth edition.^39^

### Definitions of Recurrence

Local cervical recurrence was defined as radiological evidence of recurrent disease, visualized on CT, US, or DOTATATE PET scan. Local recurrence was confirmed on cytological analysis of fine-needle aspiration biopsy (FNAB) or on histopathological analysis of reoperative surgical specimens. Distant metastases were defined as structural abnormalities on cross-sectional imaging and were confirmed on histopathological analysis of a core biopsy specimen if required.

### Statistical Analysis and Outcomes

Data were analyzed using Jamovi (The jamovi project [2022]. jamovi (Version 2.3) [Computer Software]. Retrieved from https://www.jamovi.org). Data were expressed as mean ±  SD if normally distributed, as median (range) for nonparametric data, or *n* (%) for descriptive statistics. The Student’s t test was used to compare continuous variables. Pearson’s chi-square test or logistic regression analysis was used for categorical variables. Kaplan-Meier survival analysis was used to estimate the overall recurrence and survival rate. The Cox proportional hazard regression model was used to identify prognostic factors for overall survival and disease-specific survival. The log-rank test was used to compare differences in survival outcomes. A *P*-value of <.05 was considered statistically significant.

## Results

A total of 235 consecutive patients with previously untreated MTC underwent surgical intervention in the period 1986 and 2022. Twenty-one (9%) patients were lost to follow up. The median age at presentation was 53 years (range 10-96 years), there were 133 females (57%), and the mean diameter of the primary tumor was 22 mm (SD = 18 mm). Thirty patients (13%) had germline mutations of the *RET* gene. The median serum calcitonin level at presentation was 1200 pg/mL (range 5-65 000). The demographic, clinical, and pathological data are summarized in Table 1. Twenty-one patients (9%) had distant metastatic disease at presentation, and 126 (54%) had advanced (stage III or IV) disease. Vascular invasion was seen in 91 patients (39%), and 123 (52%) had node-positive disease. Lateral CLND was performed in 100 (43%) patients, including 17 (7%) who underwent bilateral lateral compartment dissections. Twelve (5%) patients underwent prophylactic lateral neck dissection.

**Table 1.** Demographic, clinical, and pathological features of patients with medullary thyroid cancer.

	Patients (n = 235)
Age at diagnosis (years)	53 (10-96)
Female, n (%)	133 (57)
Tumor characteristics	
Size (mm)	22 (±17.7)
Gross *e*xtrathyroidal extension (%)	26 (11)
Venous invasion (%)	91 (39)
T stage	
* T1a*	56 (24)
* T1b*	57 (24)
* T2*	34 (14)
T3	62 (26)
T4a	16 (6.8)
T4b	3 (1.3)
Nodal involvement (%)	123 (52)
N0	64 (27)
N1a	18 (8)
N1b	105 (45)
Nx	39 (17)
Extranodal extension (%)	58 (25)
AJCC *s*tage, n (%)^a^	
I	84 (36)
II	23 (10)
III	15 (6)
I*v*a	88 (37)
IVb	2 (1)
IVc	21 (9)
IMTCGS *g*rade^b^	
High	11
Low	72
Preoperative serum calcitonin (pg/mL)^c^	1200 (5-65 000)
Postoperative serum calcitonin (pg/mL)^c^	12 (0-160 000)
Germline RET mutations, n (%)	30 (13)

Data are presented as mean ± SD or median and range unless otherwise indicated.

^a^Data available for 233 patients.

^b^Data available for 84 patients.

^c^Data available for 87 patients.

Abbreviation: RET: rearranged during transfection gene.

### Patterns of Local Disease Recurrence

The median follow-up time was 48 months (range 1-370). Cervical nodal recurrence occurred in 55 (23%) patients at a median time of 25 months (range 3-192) after the index operation, and 45 (80%) patients were treated with repeat surgery.

Overall, 10 (4%) patients remain under observation for asymptomatic, low-volume recurrent cervical nodal disease. Local irresectable cervical failure occurred in 2 (0.9%) patients, and irresectable mediastinal nodal disease in 29 (12%) patients, including 5 (2%) patients who presented with advanced mediastinal nodal disease. Independent prognostic factors for local recurrence included an increased risk with pathologically involved nodal status (OR 5.10, *P* = .01), and a decreased risk associated with biochemical cure after the index operation (OR 0.20, *P* = .009; Table 2).

**Table 2.** Medullary thyroid cancer characteristics as prognostic factors for local recurrence.

Prognostic *v*ariable	Hazard *r*atio (95% CI)	P-value	Multivariate *a*nalysis *h*azard *r*atio (95% CI)	P-value
*N1 disease*	10.4 (3.22-33.5)	<.001	5.10 (1.49-17.5)	.01
*Vascular invasion*	2.20 (1.28-3.79)	.005	1.45 (0.81-2.60)	.21
*Extrathyroidal extension*	3.23 (1.79-5.83)	.001	1.84 (0.97-3.49)	.06
*AJCC stage 3/4*	7.08 (3.01-16.55)	<.001	1.21 (0.17-8.59)	.85
*Biochemical cure*	0.09 (0.03-0.29)	<.001	0.20 (0.06-0.67)	.009
*M1 disease (at diagnosis)*	0.87 (0.21-3.65)	.85	N/A	
*Age >55*	0.73 (0.40-1.32)	.30	N/A	
*Germline RET mutation*	0.61 (0.30-1.23)	.17	N/A	
*Pathological high grade*	1.52 (0.34-6.91)	.58	N/A	

Abbreviation: RET: rearranged during transfection gene.

### Reoperative Surgery

A total of 65 reoperative procedures were performed in 45 (19%) patients during the study period. Two patients (4%) remain biochemically cured. Four (9%) remain under observation for low-volume nodal recurrence in the setting of other medical comorbidities, and 39 (87%) patients remain free of structural disease in the neck at the last follow-up. However, 15 (33%) have required 2 or more reoperative procedures. The median time to second nodal recurrence, and third local recurrence after initial surgery were 38 months (range 10-120) and 12 months (range 7-36), respectively. The median (range) calcitonin levels were significantly reduced by reoperative surgery (500 [14-34 600] pg/mL, and 138 [1-7560] pg/mL, respectively, *P* = .04).

Among the 45 patients who underwent reoperative lymph node dissection the most frequent site of recurrence was within a previously undissected lateral lymph node compartment. The pattern of lymph node recurrence included: previously undissected lateral nodal levels (23 patients, 51%), ipsilateral lateral nodes (8 patients, 18%), ipsilateral level 6 nodes (8 patients, 18%), previously undissected contralateral lateral compartment nodes (4 patients, 9%). Recurrent mediastinal lymph node disease was treated with reoperation in 2 patients (4%).

The rate of temporary hypoparathyroidism after revision central compartment dissection was 16%, and there were no cases of permanent hypoparathyroidism. There were no temporary or permanent recurrent laryngeal nerve palsies, and there were no major complications of reoperative surgery in the lateral compartment.

### Patterns of Distant Metastases

**Figure 1.**
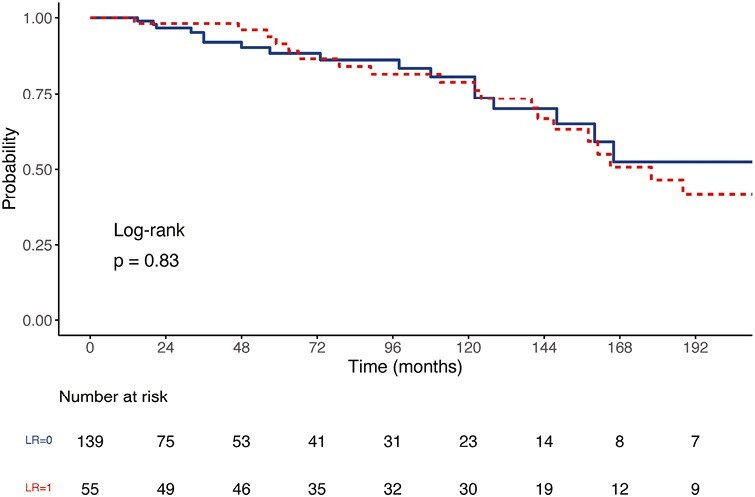
Development of distant metastatic disease stratified by local cervical recurrence. Abbreviation: LR: local recurrence.

At initial presentation, 20 (9%) patients had metastatic disease. The dominant patterns of metastatic disease included isolated bony metastases in 8 (40%) patients, and visceral metastases in 12 (60%) patients, which affected the liver (9 patients) and lung (5 patients). Overall, distant recurrence occurred in 45 (21%) patients who were M0 at first presentation, at a median time of 72 months (range 3-317). Local recurrence did not predict the development of metastatic disease (HR 0.93 [95% CI, 0.49-1.77], *P* = .83, Fig. 1, Table 3). The dominant patterns of distant recurrence were similar to the patterns of metastatic disease at presentation and included 18 (40%) patients with bony disease and 27 (60%) with visceral metastases, including 1 patient with cerebral metastases. Among patients with metastatic disease (*n* = 64), 5 (8%) underwent metastectomy for liver metastases (*n* = 3), and lung metastases (*n* = 2). Three of these 5 patients (60%) remain disease free, at a median (range) 79 (60-84) months following metastectomy, 2 of whom are being treated with selpercatinib.

**Table 3.** Predictors of development of distant metastases.

Prognostic *v*ariable	Hazard *r*atio (95% CI)	P-value	Multivariate *a*nalysis *h*azard *r*atio (95% CI)	P-value
*N1 disease*	8.82 (2.12-36.6)	.003	10.6 (2.47-45.4)	.001
*Vascular invasion*	2.55 (1.32-4.92)	.005	1.54 (0.76-3.13)	.23
*Extrathyroidal extension*	3.51 (1.85-6.66)	<.001	5.04 (2.39-10.6)	<.001
*Biochemical cure*	0.42 (0.16-1.09)	.074	N/A	
*High pathological grade*	9.1 (2.02-41.0)	.004	3.92 (0.9-17.1)	.010
*Local recurrence*	0.93 (0.49-1.77)	.83	N/A	
*Age*	1.00 (0.98-1.02)	.87	N/A	
*Germline RET mutation*	0.43 (0.19-0.97)	.04	0.50 (0.22-1.14)	.28

Abbreviation: RET: rearranged during transfection gene.

RET-specific tyrosine kinase inhibitor (TKI) therapy was associated with a longer overall survival (22 months vs. undefined median survival) (*P* = .008) in patients with metastatic disease. Thirty (13%) patients were treated with TKI therapy, including 20 (67%) with multitarget TKIs vandetanib and cabozantinib, and 16 (53%) with the RET-specific TKI selpercatinib. Five (17%) patients were treated with selpercatinib after progressing on multitarget TKIs.

Eighteen patients (8%) died from MTC during the study period, and 30 (13%) died from other causes. Among patients with metastatic disease, overall survival from the time of diagnosis of metastatic disease was shorter in patients with visceral metastases (estimated medial survival 22 months vs. 38 months, *P* = .050). One patient was palliated with locally advanced irresectable disease, before the advent of TKI therapy.

### Prediction of Survival

**Figure 2.**
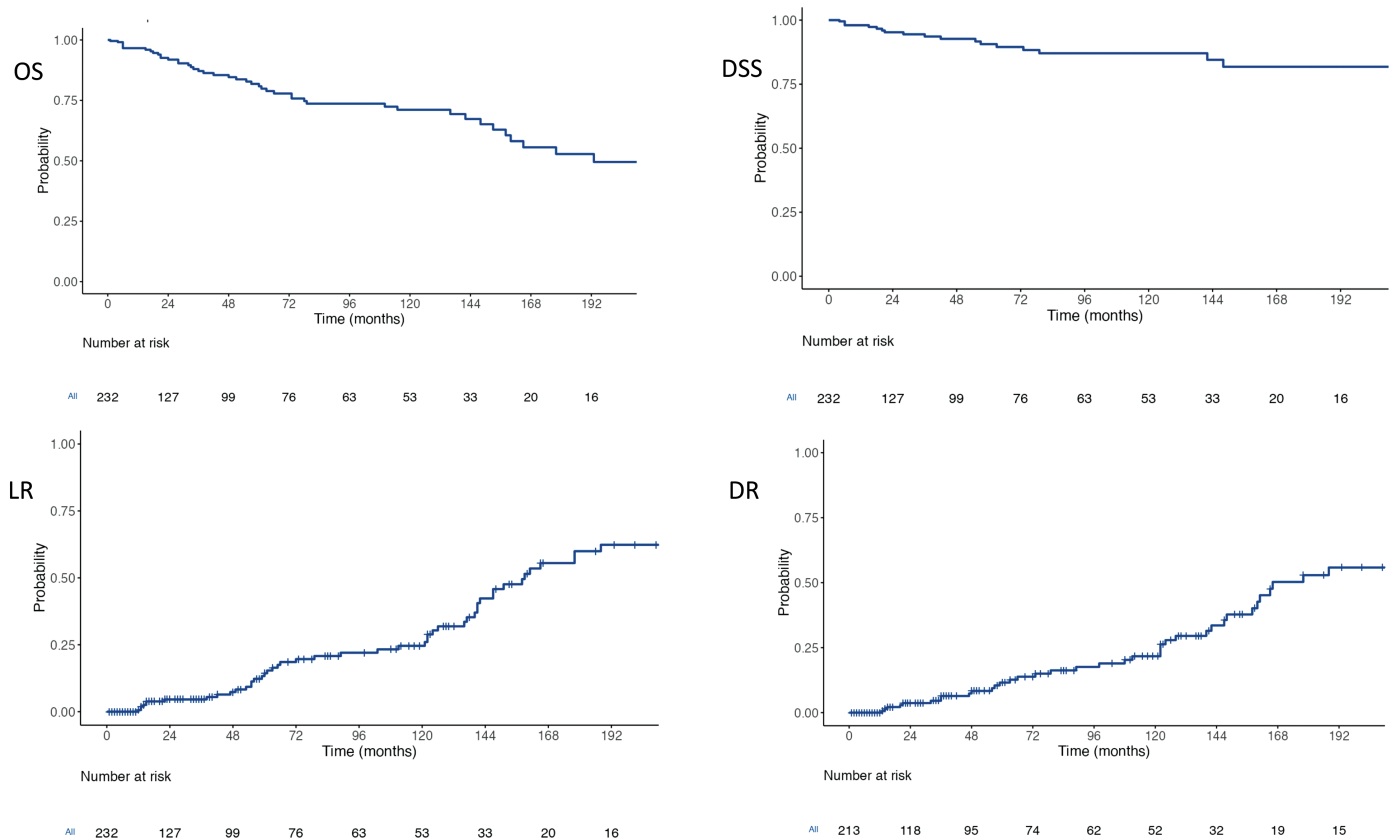
Recurrence and survival in 232 patients with medullary thyroid carcinoma after surgical management, (A) Overall survival is 82% (95% CI, 76-88) at 5 years and 71% (95% CI, 63-80) at 10 years, (B) Disease-specific survival is 91% (95% CI, 86-96) at 5 years and 87% (95% CI, 81-94) at 10 years, (C) cumulative incidence of local recurrence is 15% (95% CI, 8-20) at 5 years and 25% (16-32) at 10 years, (D) distant recurrence is 12% (95% CI, 6-17) at 5 years and 22% (95% CI, 13-29) at 10 years.

The 10-year overall survival (OS) and disease-specific survival (DSS) were 71% and 87%, respectively (Fig. 2). The predictors of OS and DSS are summarized in Tables 4 and 5. On univariate analysis for OS, nodal positivity, vascular invasion, extrathyroidal extension, high pathological grade, the presence of metastatic disease, and age >55 years predicted shorter survival. Local recurrence did not adversely impact overall survival. Similarly, failure to achieve biochemical cure was not a significant adverse prognostic factor for survival. On multivariate analysis, only high pathological grade and the presence of metastatic disease on presentation remained independently predictive. The multivariate analysis results were similar for DSS.

**Table 4.** Predictors of overall survival (OS).

Prognostic *v*ariable	Hazard *r*atio (95% CI)	P-value	Multivariate *a*nalysis *h*azard *r*atio (95% CI)	P-value
*N1 disease*	1.89 (0.87-4.12)	.11	N/A	
*Vascular invasion*	2.32 (1.22-4.42)	.01	1.32 (0.46-3.79)	.48
*Extrathyroidal extension*	2.17 (1.10-4.27)	.025	0.52 (0.10-3.89)	.52
*Biochemical cure*	0.70 (0.33-1.47)	.35	0.80 (0.33-1.97)	.63
*High pathological grade*	11.95 (3.56-40.1)	.01	13.15 (2.29-75.5)	.004
*Metastatic disease*	5.94 (2.86-12.36)	<.001	8.83 (1.39-56.1)	.021
*Local recurrence*	0.40 (0.20-0.81)	.01	0.73 (0.33-1.61)	.44
*Age*	1.05 (1.03-1.08)	<.001	1.08 (1.01-1.16)	.018
*Germline RET mutation*	0.56 (0.23-1.36)	.35	N/A	

Abbreviation: RET: rearranged during transfection gene.

**Table 5.** Predictors of disease-specific survival (DSS).

Prognostic *v*ariable	Hazard *r*atio (95% CI)	P-value	Multivariate *a*nalysis *h*azard *r*atio (95% CI)	P-value
*N1 disease*	1.64 (0.95-2.85)	.076	N/A	
*Vascular invasion*	8.75 (2.00-38.3)	.004	1.10 (0.50-14.3)	.91
*Extrathyroidal extension*	5.37 (2.05-14.1)	<.001	4.69 (0.69-33.5)	.12
*Biochemical cure*	0.83 (0.20-1.72)	.08	N/A	
*High pathological grade*	19.2 (3.66-100.5)	<.001	20.7 (2.1-205)	.010
*Metastatic disease*	15.5 (5.75-41.7)	<.001	13.6 (2.1-87.4)	.006
*Local recurrence*	0.41 (0.13-1.27)	.12	N/A	
*Age*	1.03 (0.97-1.08)	.38	N/A	
*Germline RET mutation*	0.91 (0.34-2.45)	.86	N/A	

Abbreviation: RET: rearranged during transfection gene.

## Discussion

This study examines the patterns of failure and the results of reoperative surgical management in MTC. Failure to achieve biochemical cure after initial therapy and pathological N1 nodal status are independent predictors of local recurrence, which occurs in approximately 23% of patients at extended follow-up. Reoperative surgery can be safely performed in a specialist center and is effective in achieving local structural disease control, however, up to a third may require additional reoperative procedures. Similarly, in patients with resectable mediastinal nodal disease, or oligometastatic disease of the liver or lung, metastectomy should be considered, as it often provides extended structural disease control. Local recurrence does not increase the risk of metastatic disease or death. In predicting survival, high pathological tumor grade and the presence of metastatic disease on presentation are the key independent prognostic factors.

In MTC, local recurrence or persisting disease is a common problem, occurring in up to 79% of patients at extended follow-up.^40^ In the setting of non-metastatic, locally recurrent disease, the data guiding reoperative management are limited. A correlation between survival and radiological remission has been previously demonstrated, indicating that reoperative surgery to excise recurrent disease appears to be beneficial.^41^ Our data indicate that reoperative surgery can be performed with minimal morbidity and effectively provides structural disease control, even if biochemical cure is not achieved. This includes resection of nodal disease within the mediastinum, and oligometastatic disease of the liver or lung. As systemic treatments improve and allow prolonged survival even in the setting of visceral metastatic disease,^19^ the importance of local control and the role of reoperative surgery may increase.

In predicting survival, adverse prognostic factors on univariate analysis were similar to previous reports^30,34,40,42-44^ and included nodal positivity, vascular invasion, extrathyroidal extension, failure to achieve excellent response to initial therapy and the presence of metastatic disease. In addition, the pathological tumor grade was highly predictive of both overall and disease-specific survival. When tumor grade is included in the multivariate analysis, the pathological features of vascular invasion and extrathyroidal extension lose significance, and only tumor grade and the presence of distant metastases at presentation remain independently predictive. Interestingly, excellent response to initial therapy was not independently predictive of survival. This suggests that the group of patients with persistently elevated calcitonin, but a low pathological grade is likely to follow an indolent disease course.

The role of prophylactic lateral compartmental dissection in clinically N0 patients is controversial. With the quality of contemporary high-resolution US, the benefit of prophylactic versus staged lateral compartment dissection in cN0 patients is debatable, especially when considering the increased risk of complications and the lack of proven long-term survival benefit.^14,23,24,45^ International guidelines lack consensus on this issue, with current American Thyroid Association (ATA) guidelines commenting that lateral lymph node dissection should be considered based on serum calcitonin levels,^10^ informed predominantly by the work of Machens et al who noted that preoperative calcitonin levels of >200 pg/mL were predictive of lateral lymph node metastases.^46^ More recently, Pena et al reported the safety of observation of the cN0 lateral neck, with 5% of patients developing ipsilateral lateral nodal recurrence amenable to salvage surgery at a median follow-up of 2.3 years.^22^ Similarly, Spanheimer et al noted that no difference in the cumulative incidence of distant recurrence, disease-specific survival, or overall survival between cN0 patients who were electively dissected, compared to those who were observed.^45^ Our data reinforce these findings. All cervical nodal recurrences that occurred in patients with cN0 disease who did not undergo lateral compartment dissection were amenable to salvage surgery. Furthermore, local recurrence did not influence the pace of disease with respect to distant recurrence, DSS or OS. This demonstrates the safety of a staged approach to the lateral neck in the cN0 patient. Our data support our current approach to the lateral neck in MTC: all patients undergo dedicated high-resolution ultrasonographic assessment of bilateral lateral compartments, performed by a sonographer and reviewed by a specialist radiologist. A FNAB is performed if there is suspicion of nodal involvement. If there is no evidence of disease, the lateral compartments are not dissected, irrespective of the preoperative calcitonin level. Postoperatively, calcitonin levels are closely monitored, and the lateral compartments carefully observed with serial US. In addition, we frequently utilize DOTATATE PET scan in the postoperative period if calcitonin levels suggest persistent or recurrent disease.

The main strength of this study is the large cohort of consecutively treated patients at a tertiary referral center for MTC, by highly specialized thyroid surgeons, endocrinologists, radiologists, pathologists, and oncologists, with a meticulously maintained prospective data collection process and a low proportion of patients lost to follow up. The large sample size enabled analysis of the predictors for local recurrence and survival outcomes. The main limitation of this study is its retrospective design, which impacted our ability to capture all data, especially with respect to preoperative and postoperative calcitonin levels. Furthermore, the cohort of patients treated at our center may be subject to selection bias toward more advanced tumors.

## Conclusion

Local recurrence in MTC does not predict distant failure or adversely impact survival, demonstrating the safety of the staged approach to the clinically N0 lateral neck. When recurrent disease is technically resectable, reoperative surgery can be undertaken with minimal morbidity in a specialist center and facilitates structural disease control.

## Data Availability

The participants of this study did not give written consent for their data to be shared publicly, so due to the sensitive nature of the research supporting data are not available.
